# Understanding factors affecting collaboration between midwives and other health care professionals in a birth center and its affiliated Quebec hospital: a case study

**DOI:** 10.1186/s12884-017-1381-x

**Published:** 2017-06-26

**Authors:** Roxana Behruzi, Stephanie Klam, Marleen Dehertog, Vania Jimenez, Marie Hatem

**Affiliations:** 1 Department of Family Medicine, McGill University/ The Research Center of the CISSS at Outaouais, 5858, Chemin de la Côte-des-Neiges, Montréal, QC H3S 1Z1 Canada; 2 Department of Obstetrics and Gynecology, McGill University, Montreal, Canada; 3 Maison de naissance Côte-des-Neiges, CIUSSS Centre-Ouest-de-l’île-de-Montréal, 6560 Chemin de la Côte-des-Neiges, Montréal, QC H3S 2A7 Canada; 4 School of Public Health, Department of Social and Preventive Medicine, Université de Montreal, 7101 Avenue du Parc, Montreal, QC H3N 1X9 Canada

**Keywords:** Interprofessional, Interorganisational, Collaboration, Midwives, Birthing center

## Abstract

**Background:**

A better understanding of the processes of collaboration between midwives who work in the birthing centers, and hospital-based obstetricians, family physicians and nurses may promote cooperation among professionals providing maternity care in both institutions. The aim of this research was to explore the barriers and facilitators of the interprofessional and interorganizational collaboration between midwives in birthing centers and other health care professionals in hospitals in Quebec.

**Methods:**

A case study design was adopted. Data were collected through semi-structured interviews with midwives, multidisciplinary professionals and administrators, through direct observation of activities in maternity units and field notes, and a variety of organizational and policy documents and archives. A qualitative thematic analysis method was used for analyzing transcribed verbatim.

**Results:**

The study suggests the close intertwinement between interactional, organizational and systemic factors in regard to barriers and opportunities for collaboration between midwives in birthing centers, and physicians and nurses in hospitals in Quebec. At interactional level, our findings show a conflict in scope of midwifery practice, myth about midwives, pre-judgment, and lack of communication skills between health care providers in the studied birthing center and hospital. At the organizational level, this investigation shows that although midwives have complete access to the hospital with which a formal agreement was signed, they were not integrated in hospital because of lack of interest of midwives and differences in philosophy and scope of practice among healthcare professionals as well as the culture of organizations. At a systemic level, in spite of excessive demand for midwifery care, there are not enough midwives to cover these demands.

**Conclusion:**

Maternity care professionals require taking a collaborative approach in working and the boundaries of responsibility need to be redrawn. The inter-professional collaborative work between midwives and other maternity care professionals is crucial to improve access and women’s choices for maternity care in Canada. Although having collaborative and multidisciplinary teamwork is a goal of maternity care systems, it is hard to achieve.

**Electronic supplementary material:**

The online version of this article (doi:10.1186/s12884-017-1381-x) contains supplementary material, which is available to authorized users.

## Background

The need for multidisciplinary maternity care and interprofessional collaboration is obvious, as it helps to solve the shortage of care providers in the maternity care system, and guarantees improvement in maternity care services in rural areas, in Canada [1]. Midwifery is a new profession in Canada and midwives attend about 2 to 5% of births, depending on the provinces. Collaboration between midwives and other maternity care professionals assures continuity of care, and better outcomes for both women and newborns [2]. Collaboration is an active process requiring the will to make meaningful contact between people [3]. D’Amour et al. [4] highlighted some key concepts in interprofessional collaboration such as: sharing, partnership, interdependency, and power. Bronstein [5] identifies several constitutional factors that influence collaboration in practice such as: interdependence, shared ownership of goals, and flexibility toward collaboration. Lemieux-Charles et al. [6] suggested that the type and diversity of clinical expertise involved in teams affects the improvement of patient care and organizational effectiveness. Collaboration, conflict resolution, participation, and cohesion were the factors most likely to influence staff satisfaction and perceived team effectiveness.

Interprofessional collaboration requires a variety of skills and competencies as well as a variety of contributions from professions and organizations [7]. The ultimate aim of collaboration is to integrate services at the point of delivery. This integration has to be taken into account at the individual and interpersonal as well as organizational and interorganizational levels [7]. On the one hand, individual factors such as skills, competence, and work experience can serve to support various interprofessional networks and teamwork. On the other hand, the structures of the organization- for example, the degree of formalization, distribution of resources, competition, management and leadership governance- can also have an important impact on the interorganizational dynamic and coalition [7].

Maternity care practice is characterized by close co-operation between obstetricians, midwives, family physicians and nurses, nevertheless, conflict has been reported between many healthcare professionals. The level of conflict or co-operation between maternity care professionals depends on the organization and culture of the working environment [8]. Collaboration in midwifery terms has been defined as: “exercising of effort by midwives and physicians towards each other for the purposes of sharing functions, rewarding and effecting care to women and their families.” [3, 9] The term collaboration is used with related terms such as co-operation and teamwork [10].

Despite much progress in the legitimization of the midwifery profession in Canada, there are still disagreements among maternity care professionals regarding midwifery autonomy and midwifery practice. Reducing conflicts and enhancing collaboration has benefited the majority of women with both low and high-risk pregnancy [3]. In collaborative climates, women feel confident about the birth choices they make. A full integration of midwives into the Canadian healthcare system needs removal of conflicts, barriers to collaboration and obtaining hospital access privileges. The poor integration of midwives into hospital comes partly from the difference between midwives’ and physicians’ philosophies of care [11], as well as hospital policies that place varying degrees of control and restriction over the way midwives provide care [12].

The Quebec 2008–2018 Perinatal Policy foresees midwives will be responsible for 10% of the prenatal care and births in Quebec by 2018 [13]. This policy puts pressure on the collaboration between the *Centre de Santé et de Services Sociaux* (CSSS) to which midwives belong, and professionals in hospitals. This collaboration guarantees women receive continuity of care and access to different services and professionals in hospitals [13]. Different philosophies of care among maternity care providers- especially midwives versus obstetricians- may lead to poor communication, tension, or even rivalry. One may question the nature of interprofessional collaboration between midwives in birth centers and other professionals in the hospitals in Quebec several years after the release of the Quebec Perinatal Policy in 2008.

Limited research exists on interprofessional and interorganizational collaboration between midwives and other healthcare professionals in Quebec, Canada. From previous literature, we have some knowledge of diversity of the attitudes, practice and collaboration of maternity care professionals. We still need to develop a better understanding of this collaboration in order to become aware of potential barriers to this collaboration [1, 14, 15].

The aim of this paper is to explore the factors that influence the interprofessional and inter-organizational collaboration between midwives in a Quebec birth center and its affiliated hospital.

### Conceptual framework

A few studies have already explored collaboration from a conceptual or theoretical perspective [4, 5]. An effective inter-professional collaboration emerges from a dynamic interaction between organizational and personal characteristics. Amour et al. [4] examined the conceptual basis of inter-professional collaboration. Authors highlighted sharing, partnership, interdependency, and power as key concepts in inter-professional collaboration. A similar review by Bronstein [5] identified the interdependence, shared ownership of goals, and flexibility as constitutional factors to collaboration, while a history of effective collaboration attributes as influencing factors to collaboration. Lemieux-Charles et al. [6] suggested that the type and diversity of clinical expertise involved in health care team have impact on the improvement of patient care and organizational effectiveness. Collaboration, conflict resolution, participation, and cohesion were most likely to influence staff satisfaction and perceived team effectiveness [6]. Trusting and mutually respectful relationships might create positive feedback into the maternity care professional. The professional rivalries and philosophical differences over childbirth practice generate significant tensions in the work place.

**Fig. 1:**
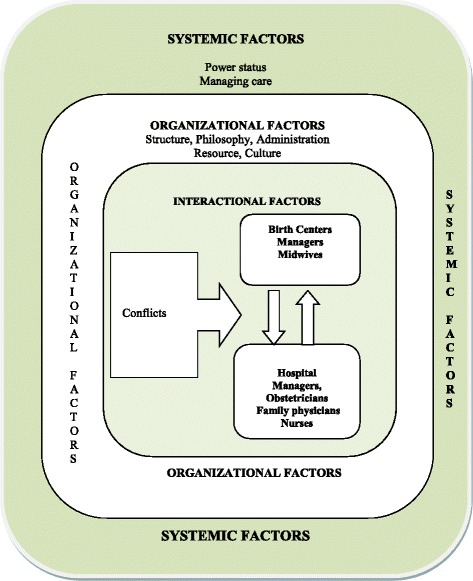
Conceptual framework: the determinants of collaborative work between midwives in a birthing center and other maternity care professionals in affiliated hospital to birthing center

The conceptual framework of this project has been constructed based on “determinants of successful collaborations between healthcare teams” described by Rodriguez. These determinants include interactional, organizational, and systemic factors [16]. The midwives in birth centers and other maternity care professionals in the hospitals affiliated to birth centers are supposed to have a mutual collaborative relationship. This relationship, however, is often influenced by interactional factors, such as *interpersonal trust, mutual respect, and open communication*. On the other hand, conditions within the organizations themselves, such as *organizational structure, leadership, philosophy, team resources, and administrative support* also have an influence on the interprofessional co-operation and collaboration between the professionals working in birth centers and hospitals. Finally, collaboration between professionals in birth centers and hospitals is under direct impact of their external environment, such as *social factors.* (See Fig. 1).

## Methods

### Design

A *single-case study* was performed. The phenomenon under investigation, *“interprofessional and interorganizational collaboration,”* represents a complex phenomenon making it more sensible to choose a research design that allows taking a meticulous and more holistic view of the case. We did an embedded case study [17] since particular attention was paid to people in the organization at different levels of ‘administrative’ and ‘professional’.

### Case

The case under study was a birth center (“Maison de naissance” in French) that signed an agreement with a tertiary university hospital, in 2005. The birth center was the first of its kind established in Montreal, with about 300 births per year. The chosen hospital was the second setting in Montreal allowing midwives to deliver babies there. The choice of setting was influenced by the accessibility on-site to the investigator.

### Participants

The sample was target people from different administrative and professional levels of practice at the hospital and birth center. We chose a purposive sampling framework and individuals were recruited for specific key characteristics [18]. The purposive sampling included: 1) administrators in both hospital and birth center, 2) midwives with different levels of experience, 3) nurses at different levels of practice, training degree and level of experience, 4) the obstetricians & gynaecologists, 5) the family physicians.

### Data collection

Various sources of data collection were used to establish the construct validity and reliability of the case study and perform the triangulation of the data sources [17]. The data collection period was from Jun 2011 to Oct 2014 and included:

1. *Semi-structured interviews:* The interviews took place until saturation occurred. The duration of the interviews was approximately 60 min. The semi-structured interviews were performed with four administrators, two family physicians, five obstetricians and gynecologists, nine nurses, and five midwives. The questions were categorized into three groups: I) Questions related to interactional factors such as interpersonal trust, respect, and open communication; II) Questions related to conditions within the organizations themselves, such as organizational structure, leadership, philosophy, team resources, administrative support, co-ordination and communication; and, III) Questions related to external environment, including social and cultural factors*.* (See Additional file 1)
  The interviews were performed in both English and French. As the investigator was bilingual, she translated the French interviews to English and these were checked by the interviewees to ensure accuracy before performing analysis.
2. *Participant-observation and field notes:* Fieldwork took place between July 2011 and October 2014. The observations embraced what people do, what they value, the social interaction between them, and the circumstances around birth units in the hospital. The observations also focused on interprofessional and/or interorganizational activities, events and conversations in the birth unit of the hospital, as well as the interdisciplinary meetings and workshops in the hospital. To complete the observation and field visits, the principal investigator attended in total of eight More-OB workshops, More-OB Core team meetings, and multidisciplinary maternity team meetings. Field visits were performed at least 3 days per week during the period of data collection. The observations continued until saturation of data was achieved.
3. *Documents and archives* included: 1) the administrative document regarding rules and regulations for transfer of midwifery clients from the birth center to the hospital, 2) documents relevant to birth center rules, regulation and mission, 3) multidisciplinary obstetric workshops documents and modules 4) administrative agreements between the birth center and the hospital, and 5) the minutes of meetings that were considered to be relevant to our study. The documents were collected with approval of the administrators. The information was gathered through a systematic search for any pertinent document, and through field visits.

### Data analysis

A qualitative analysis was performed on the transcribed interviews, field-notes, as well as the documents. We used *thematic content analysis* with both *deductive and inductive* approaches. Thematic analysis is a widely used qualitative data analysis method that focuses on identifying patterned meaning across a data set. In thematic analysis, frequencies let researchers interpret or “let emerge” themes from the corpus. Thematic analysis describes data set in rich detail. It can also interpret various aspects of the research topic [19]. Our deductive or theoretical thematic analysis was driven by our theoretical framework and in concordance with Rodriguez’s determents of successful collaborations between healthcare professionals: “*definition of collaborative work, interactional factors, organizational factors, systemic factors.”* Our subthemes were identified in inductive thematic analysis and were strongly linked to the data themselves. The subthemes include: “*conflict, philosophy and mission, administration, resources, culture, structure, power and status, and managing care.”*

Considering the Braun and Clarke [20] thematic analysis guideline, the investigator performed six phases of analysis. She started with immerging herself in the data by transcribing verbatim. Next, she undertook reading of the transcribed data in an active way, while searching for meanings and patterns [20]. Then, she generated initial codes. Our coding approach depended on the data itself and it was coded around specific questions, while it was in concordance with Rodriguez’s determents of successful collaborations between healthcare professionals. Next, the different relevant codes were sorted to form an overarching theme. Some initial codes formed main themes, whereas others formed subthemes; still others constructed categories. After reviewing and refining themes and ensuring that those themes appeared to form a coherent pattern, they were presented for analysis of the data within them. *QDA Miner version 4.0.11* computer-assisted qualitative data software was used to manage data.

## Results

In total, 25 healthcare professionals participated in this study. The mean age of participants was 45 and ranged from 29 to 64 years. The mean experience of participants was 12 years and it varied between 1 to 20 years. Four major themes emerged from data. The first theme is the definition of collaborative work. The other three major themes and subthemes highlight significant factors in interprofessional and interorganizational collaborative work. (Table 1)

**Table 1:** Factors affecting collaboration between midwives and other health care professionals in a birthing center and its affiliated Quebec hospital

Themes	Subthemes	Categories
Definition of collaborative work		
Interactional factors	Conflict	Conflict over Professional Philosophy
		Conflict over Autonomy, Professional Territory, Work Style
		Conflict over Compensation Issue
Organizational factors	Philosophy and Mission	Hospital versus Birth Center Philosophy
	Administration	Lack of Midwives at the Administrative Level
	Resources	Dedicated Financial Resources
		Essential Infrastructure and Time
	Culture	Culture of Team Work
		Culture of Interventionism vs. Non-Interventionism
	Structure	Organizational Rules and Regulations
Systemic factors	Power and status	
	Managing care	

### Definition of collaborative work

Most of the participants agreed on the benefit of working with others for the well-being of the patient. Collaborative work was defined as *“working together for the same goal, working with agreement, having a good communication and exchange of ideas, listening to each other, meeting the needs of other professionals,* and *being available to each other.”*

We are only here for the patient’s sake, for the well-being of baby and mother. Even when I disagree with the way others are practicing, I still have to collaborate with what they are doing for the well-being of the mother and the baby. (Nurse 7)

You have to often work together, not just in looking after a specific patient, but working together for developing agreements and protocols, and making the system flexible to accommodate all the needs for everybody who is looking after pregnant patients. (OB4)

The family physicians defined collaboration as “being respectful in communication with people,” and *“being constructive in discussing issues, not criticizing them.”*

If you have problems, you focus on the problem, not on the people and if you have positive thing, you say it to people, and you respect the people for their professional talents and skills that are different from what you have. (PH2)

### Interactional factors

#### Conflict

Most of the conflicts raised between maternity care providers were around professional philosophy, autonomy, professional territory, work style, and monetary compensation.

##### Conflict over professional philosophy

Most of the conflicts between obstetricians at hospital and the birth center midwives arose from different philosophies of care and the way they were practicing. Midwives gave a lot of importance to “*continuity of care*, *empowering women*, and *one-on-one care.*” They believed that such a practice was not happening at the hospital and that obstetricians do not give enough credit to individualized care:

Our philosophy is very different, so sometimes it is difficult to understand each other. For us, it’s important that the woman be part of the process, but their protocol and the way they work (obstetricians) don’t put the woman in the center of the care …and they don’t necessarily believe or adopt a normal birth. Sometimes we have the same situation but they would do something different. (Mid 1)

The obstetricians expressed their belief in another way:

“Their agenda is vaginal delivery at all costs, and that’s not my attitude towards birth. So you obviously have two very different attitudes towards birth”. (OB3)

One of the nurses believed that it would be good to have midwives in the hospital but they would have to change probably a little bit of their own philosophy. (Nurse7) Most of the nurses defined obstetricians’ management of labor as “*very pro-active*” vs. midwifery management of labor in a way to “*let nature take its course*”. The nurses also defined their care as an *“interventionist*” and “*invasive*” one. The analysis of data showed there were difficulties in finding common ground between midwives and obstetricians around interventions. The obstetricians mostly saw pregnancies as a high-risk event and some had never witnessed a normal pregnancy and a normal delivery without intervention.

We like to prevent problems before they arise, or detect things early and intervene more early when things can still be done, and when the risk of complications is lessened….so it is a different philosophy, a different approach. (OB2)

By contrast, the midwives considered everything as normal until something comes up:

Pregnancy is a healthy issue in the life of a woman and we try to be preventive and try that things go on a good way… they (obstetricians) are coming from the vision when they were trained like “ok there's something wrong and so we have to cure it”. (MidA)

##### Conflict over autonomy, professional territory, work style

Most of the obstetricians did not perceive any major power struggles between themselves and midwives and were not involved in, nor witnessed any conflict. Two obstetricians however criticized midwives saying, “They (midwives) want to decide when it is time to call a doctor.” There was willingness from the obstetricians to work collaboratively with midwives in a hospital setting. The participants, however, believed that in such a context, the collaboration would most likely be influenced by the obstetricians, thus restricting the power and autonomy of midwifery practice:

Obstetricians would have said something like “ok…you have to work like we do”, and the midwives didn't want to have to be told that. (PH2)

The obstetricians expressed that the midwives do not want to be integrated to hospital and they do not want to become part of a team, as they prefer working independently in a birth center:

I think the hardship is that they have a set of expectations and they really feel that the midwifery experience should be a separate experience from the hospital. (OB5)

The administrators at both the birth center and the hospital emphasized that midwives would rather not come into a hospital setting, because they know that the environment of the birth center is a better match with the values of their practice and how they see pregnancy and birth:

In Ontario, midwives have hospital privileges, but the way Midwifery Authority in Quebec built the profession here is not like Ontario and Quebec decided that midwives would not have privilege in hospitals. Now we can go to the hospital to have hospital births under our responsibility but it is not a privilege. (Mid1)

In effect, the midwives were not sure what are the advantages of having hospital privileges because they already have access to all the services that other professionals have in the affiliated hospital. Most midwives desired to work autonomously in order to protect “natural and physiological birth” as well as sustain the client’s desire for giving birth out of hospital:

It is rare that women ask to have a hospital delivery with a midwife. Most of them – even if they had chosen to have a hospital birth at the beginning of the pregnancy – during the pregnancy are going to change their idea and they ask for birth outside the hospital... If no women wanted to have birth in a birth center, I would agree to go work in the hospital setting, but they do not. They want to have the delivery at the birth center. (Mid2)

I am tired of hearing: “Midwives should go in the hospital”. Yes, but the population comes to see us because they want to give birth outside of the hospital. I will follow the women to where ever they want to go, but if they come to see me, it is not because they want to go inside the hospital. Here, we offer women the possibility to go to the hospital and we have, I would say, three births a year from women that decide to go there with us. (Mid1)

Most of the nurses did not support the ideas that midwives should work in hospitals as hospital staff:

I have never really thought of it, that if they would be here as a colleague, I do not know how it would be; I think a lot of people would not like it. […] We run the department and we are nurses so whenever the midwife come (for transfer), it is like “now the patient is here, she is mine, you have no say”. Yeah, I think a lot of people think that way, … maybe because they feel threatened of having the other one’s opinion. (Nurse 3)

Some of the nurses stated some disagreement between the midwife and the nurse especially on particular subjects like patient demand for an epidural:

This becomes a particular issue when the patient asks for an epidural, and the midwife says “no, don’t take it now”; so I can see some nurses reacting like this: “the patient wants an epidural, don’t interfere” or “she’s the one who really has a sense of what she's going through so she should be able to decide” which is true. (Nurse 7)

There are personalities in nurses that can be confrontational, that can be disrespectful to the process, or disrespectful to the fact that the midwife and the patient have this bond, so it is really the individual personalities that can make it an unpleasant experience for patient. (Nurse4)

##### 3.2.1.3.Conflict over compensation issue

The nurses and family physicians believed that midwives could become a potential rival to obstetrician, and that clients may increasingly chose midwives over obstetricians for routine, low-risk prenatal care and delivery:

They (obstetricians) are afraid of losing part of their practice to the midwives, which I find is sad because they have much more to do than take care of a low risk pregnancy. (Nurse 6)

Last year, I was about to resign… because they (obstetricians) were telling us we had too many patients that we were delivering. We had to cut back our numbers, while we are not even doing a quarter of the births in the hospital and there is no way to negotiate [] so they do not need the family doctors; family doctor is non-sense, because it takes away dollars from them! (PH1)

The family physicians believed that midwives and family doctors should be doing primary care and obstetricians should be doing the secondary and tertiary care and dealing with the complications, but obstetricians want to do the primary care because they are being paid “fee for service”. The family physicians commented on the possible way of changing the remuneration system in hospital from “fee for service” to a “forfeit” or “fixed price”:

There should have to be no fee for service! They (obstetricians) would have to be paid a salary when they are on-call and … then they would not be so much interested in doing a volume. (PH1)

One of the administrators also referred to the earlier resistance at the time of legalization of midwifery in Quebec:

The physicians in hospital settings were afraid when they said, if the government invest in the first line and in the birth centers, then we will lose a part of the money”. (MidA)

One family physician also referred to the president of the Québec Medical Association’s concerns about midwifery professionals at the time midwifery was legalized in Quebec territory:

The Québec Medical Association was warning everybody of what a threat the advent of midwives was to family doctors and to obstetricians, that they were going to take away the work, and take the place of medical professionals, and that this was a bad thing! (PH2)

### Organizational factors

#### Philosophy and mission

##### Hospital versus birth center philosophy

The hospital and birth center’s philosophies both focused on patient-centered care but had many differences between the two organizations and their individual philosophies.

According to the administrators and nurse participants, the hospital philosophy and mission were defined as *‘care by all, for all’* (Adm1) and taking the patient and the family into consideration (Nurse4). They mentioned that the core strength in the hospital was team-work:

There are high volumes of patients, and it is only by working together that we get through taking care of all those volumes of patients. (Adm1)

The nurses felt that in spite of their differences, all maternity care providers, including midwives, were working together towards a common goal that was patient safety and well-being:

We are working together; the midwife is there for the patient’s support and I am doing the nursing part because (the patient) is under doctor’s supervision and the doctor is looking after her. I do not feel there is much difference. (Nurse 7)

We are all working for the same goal; for a healthy delivery, healthy patient, healthy baby… so we do not have any choice. I am going to be honest again… I do not like working with some people, and I am sure some people do not like working with me, but we have to keep our differences outside and just say ok, we’re working for the well-being of the patient’. (Nurse 8)

The philosophy surrounding midwifery care in the birth center was centered on empowering women and allowing birth to be as natural as possible while placing the patient at the center of the care:

It is certain that the greatest philosophy of midwifery is to empower women in their process of childbirth….so women make decisions relative to interventions they want or not. (Mid3)

The midwives wanted to be more autonomous and “to protect more natural birth:”

Ok, we believe that women are able to take charge of their own pregnancy and delivery; we believe in empowerment; yes, we believe in the capacity of women to deliver their own babies physiologically. (MidA)

The obstetrician’s primary goal was focused on the safety of mother and baby. Some of them had critical views towards midwifery and the “natural birth” approach:

So our goal is simply safety of mother and baby, and their (midwives) goal is that “it has to be a vaginal birth or it is a failure”. We do not look at it that way. (OB3)

### Administration

#### Lack of midwives at the administrative level

The lack of midwives at the administrative level was considered to be a barrier to collaboration with other professionals. MidA commented that there is a need to have midwives who know the maternity field very well and have leadership skills:

You have to put those people in directive positions and then you can go to action…We do not have midwifery representation at the level of the ministry []… In 2005, when the new building for the social and health service came out, midwives were saying that it’s important to have representation from other professionals … ideally, you put a regional midwife to show how to implement midwifery care in the region! (MidA)

Midwives and nurses pointed out that “having a spirit of collaboration” is the best facilitator of interpersonal collaboration. The MidA emphasized the importance of inviting midwives to the “Tables de Concertation” to help to implement policies. MidA believed that most “decisions are made by people who don’t really know how a birth center works, so they make decisions about things that they don’t know.” (MidA)

Each person is bringing his/her point of view and expertise and so it is important to have all the people that are necessary to create a good plan. That is the reason why sometimes you can have misunderstanding and poor collaboration because decisions at the higher levels are not sound, then the lower levels are not able to follow. (MidA)

Many nurses revealed that a vision of promoting interprofessional and interorganizational collaboration does not exist between midwives and other professionals at the administrative level in their hospital:

I don’t feel that there’s someone really in a management position who is really interested in moving that (collaboration) forward. (Nurse 1)

### Resources

#### Dedicated financial resources

The administrators emphasized the importance of having some protected budget given by government to CLSC (Local Community Services Centre) and hospitals for implementing Quebec Perinatal Policy and expanding midwifery practice. The hospital administrators complained about a lack of resources devoted to midwifery student training:

If the government dedicates resources for training midwives and incorporating midwives into our multi-disciplinary team, it would be very welcome. (Adm1)

According to the administrators, the resources they had were not enough to offer midwifery clients more than what was already in place. Moreover, there were always competing demands on resources:

Right now, resources that are supposed to be going towards maternal child health are going to patients in the emergency department and people who are over the age of seventy. So we're competing within a fixed resource pie and that's where it becomes quite difficult and challenging. (Adm2)

The MidA mentioned “most of the time money is invested in hospital settings and not really in the first line care”. She complained about lack of attention to the first line care and lack of management of care providers:

Obstetricians have been studying for 10 years and it costs thousands and thousands of dollars to society, and then they spend 60–70% of their time (taking care of) low risk births. Some countries do a better job respecting first, second and third line care and they have very good outcomes, even better outcomes than we have. (MidA)

#### Essential infrastructure and time

Most of the participants complained about lack of time for interaction and communication. The nurses pointed out that they do not have time to get together once a week to do formalized teaching on a topic, review a case, or discuss different issues.

Even just to discuss how we could improve issues with a certain doctor, or maybe we are having issues with the midwives… we do not have time and I think that is a shame! (Nurse5)

No! Here, we do not have time, because we are on-call one day, and then we are running all over the place after! (PH1)

One of administrators (Adm4) mentioned that the physicians only have time to go from room to room, because they provide care for maybe a dozen patients at once:

Everyone is very busy in their schedule…I have a hard time getting nurses to a one-hour teaching session. How am I able to relieve them from their duties… to come and meet with midwives for the goal of collaboration, when I have so many other patient safety priorities that need to be met at the same time? (Adm4)

The nurses and family physicians also criticized that there was not enough space for appropriate interaction with other care providers:

The nursing station is too small and we are always outside like around… standing. We do not have that space to interact or talk or ask questions or things like that. Even for the doctors it is very difficult. (Nurse6)

When we asked about the possibility of transferring more midwifery clients to hospital in case it would be needed, the obstetricians were concerned that the hospital is overwhelmed in terms of accepting deliveries and no new infrastructure in hospital is reserved for midwifery clients. (OB2 & OB3)

Well the problem is the shortage of resources… we cannot increase our numbers any more than we have [ ] it’s a problem that the Minister of Health has been offering solutions like midwives, but have not gone ahead and made any actual changes to the care that we provide; they have not increased the budget, they haven’t increased the beds! (OB5)

The proximity of the birth center to the hospital was seen as advantageous only in terms of “making it easier to meet” to solve problems, but participants did not think it makes any difference in terms of collaboration or improved safety for patients:

It could be a hundred miles away; it does not make a difference. Although it is physically close by, we do not go there and they do not come here unless they have to transfer a patient here. (OB1)

The moment that you have to call an ambulance and step into a car to come to the hospital –I do not care if it is three block or three kilometers – it is the same process. (OB4)

Because we communicate mostly by phone, we do not really see the midwives. They phone us when they need to transfer a patient; we do not phone them because we do not have patients to send to them! (Nurse5)

### Culture

#### Culture of team work

The multidisciplinary professionals in the hospital as well as midwives who were working in the birth centers pointed out that they are motivated to practice uniformly, function as a group, and support each other. The participants found it to be a facilitating factor in terms of collaboration within and between professionals and organizations:

This hospital has a culture of collaboration: people do work together and get along more than other hospitals. It is a special environment. So, I think the corporate culture of this place is to work together in a collaborative way. (OB3)

In this birth center, the accent is on real teamwork, and each team helping others. (MidA)

Some nurses said they have a culture of informal communication that is not the best for all. One of the nurses believed that their culture definitely impacts on the interactions they have with midwives (Nurse4). She stated that sometimes midwives are surprised about the nurses’ communications:

We are quite informal with each other, so I think that sometimes there is a slippery slope because we can be too informal and then people may feel disrespected. I think that there can be a fine line between being buddies with a doctor or with a midwife, and then slipping into “Oh! That was actually disrespectful that comment that was made”. (Nurse 4)

#### Culture of interventionism vs. non-interventionism

The greatest obstacle seen towards interprofessional and interorganizational collaboration by all the participants was the nature of the tertiary teaching hospital and its highly interventionist environment versus a culture of noninterventionism in the birth center:

Because it is a high-risk center, I find we are very prone to interventions, inductions, C-sections and things like that, then yes in a certain way it could influence the role of the midwives here, because we are so interventionist. There are a lot of interventions being done whereas in regards to midwife’s environment, there are usually no interventions. (Nurse 3)

When you are in a hospital, the epidural is just in place. You know, almost every mom when they hit transition phase, will ask for something for pain, an epidural like “I can’t do this… I need pain medication, you're torturing me”. When you are at a birth center, you know that, that option requires a transfer to another institution and is more complicated. So you’re going to be more likely to pull up your boot-straps and do what you need to do to get through the delivery. (Adm4)

On the other hand, while the nurses in the hospital pointed out their obligation to follow common hospital protocols like “I’m an employee at the hospital and I have to follow its policies”, some of them emphasized their own willingness to follow the patient’s plan if it was possible. One of the nurses highlighted that even if they come from two different angles and they are more intervention-orientated, it does not mean that “they wouldn’t help a patient who doesn’t want to have an epidural”. (Nurse 7)

The obstetricians felt pressured sometimes to accelerate deliveries to free more space for new patients:

We will maybe accelerate the labor more; maybe break her water a little bit faster because we need to get patients moving. You have to manage the whole case room, you want to do what's best for the patient but you need to move patients…you don't want them to be dragging on too long … there’s more patients that keep coming, so you need to get patients delivered. (OB2)

The family physicians referred to the hospital culture as high-tech-interventional-no-support, however, they considered themselves between the two groups of midwives and obstetricians in terms of birth practice:

We do not believe that high-tech is better than low-tech, and so that is why say, we are kind of hybrids between the midwives and the obstetricians; we can do the high-tech, but we know that it is actually destructive to use high-tech when low-tech will do them same. (PH1)

The family physicians believed that the hospital care providers are under much pressure all the time so that they have forgotten “the art of supporting or the touching”. According to family physicians (PH1) nurses in hospital have not been trained to support women in labor:

The nurses are never told, “Why don’t you go and support a woman”, they’ve always being told “why didn’t you chart this, why did you stop the Syntocinon?” No one even explains to nurses the value of supporting a woman, no one tells them that if you're with the woman in the room, you can avoid the epidural, and avoid the intervention, so you can see how it can be a little bit of a clash sometimes, the culture of midwives with their supporting to avoid the interventions, and nurses who are trained to do interventions and not to avoid them. (PH1)

On the other hand, the nurses showed their willingness to support their patients in labor but complained about the lack of time for doing it:

Even if we would like to support the patient we cannot, because we do not only have one patient; we have two or three patients at one time. So when we have a patient in labour – the patient who doesn’t want an epidural – and you have another patient… it's hard for us, in the sense that we have to be running around going… so the one- to- one caring – as a nurse – doesn’t exist. (Nurse 8)

The midwives supported their culture of reducing unnecessary interventions in obstetrics but they were very cautious of the importance of C-section or other necessary interventions:

In birth center, there is a culture of non-intervention. It is fair to say that pregnancy for us is normal, but it is not at all costs! (Mid3)

### Structure

#### Organizational rules and regulations

The participants revealed that the differences in the structure of the hospital and the birth center and their organizational rules and regulations might influence the nature of collaboration between maternity care professionals in those organizations. Midwives had their laws and regulations called “Regulation on consultation and transfer of clinical responsibility to a physician” and they had to follow all the conditions for when they must make a transfer or consult [21].

The hospital professionals revealed that the hierarchical nature of the hospital with the doctors in influential positions represent barriers for interprofessional and interorganizational collaboration. The nurses mentioned that “doctors have the power; they are the ones who decide the last word” (Nurse7). The family physicians also believed that the obstetricians have a lot more power than family physicians. One of the administrators said:

Nurses end-up taking doctors’ orders. We can negotiate or say that we do not agree but at the end of the day, what the doctor says goes. You know the nurse can refuse to do something that she does not feel is safe or comfortable doing, sure, but she cannot change what was prescribed... Of course, there is hierarchy there... Whereas the way the system is set-up, midwives do not take orders from doctors… Once you bring midwives into hospitals, who is at the top of hierarchy? Doctors! (Adm4)

The midwives demonstrated their autonomy and confidence while working in the birth center and felt that they cannot provide the same care under the control of doctors in the hospital.

### Systemic factors

#### Power status

Both birth center and hospital administrators expressed the importance of having midwife representation at the government level in order to attempt to influence decisions and to provide more support and resources for midwives:

I am involved in lobbying government to provide more support to nurses, and I think that government needs to be influenced to meet the needs. If the government is going to issue a policy, saying that midwives are going to deliver ten percent of the babies in the province of Quebec, then there needs to be lobbying of the government to provide the resources to improve that interprofessional collaboration. (Adm2)

In Ontario, a midwife is in charge of the whole department of midwifery at the ministry of health level. That would be helpful if you have somebody who is sitting on the top level and understands midwifery. (MidA)

### Managing care

The administrators emphasized the importance of collaboration and openness of professionals to guide women towards appropriate healthcare providers.

An obstetrician follows three hundred pregnancies in a year! The obstetrician should prioritize the difficult pregnancies; and say to the woman: “I could suggest to you to find a midwife or to find a family doctor” and say that “I work in collaboration with those professionals, and if you develop a complication in your pregnancy, they will call me”. (Adm4)

The administrators agreed that women should be informed about their choices regarding care providers and the importance of directing women to specialists for high-risk pregnancies. (Adm2)

The CLSCs have a big role to play because they see the woman in the early stage of the pregnancy. Some of women do not even have a physician or someone to follow their pregnancy, and if the CLSCs could explain clearly the role of each one and the choices that are available for the woman, I think that would be a nice help. (Adm2)

On the other hand, the administrations believed that their resources are not being adequately utilized and most obstetrician clients are low-risk mothers:

I think obstetricians should handle more high-risks patients and I think family practice doctors and midwives should be handling more low-risks patients. (Adm4)

## Discussion

This study provided firsthand opportunities for exploring the nature of work between maternity care professionals in Quebec and the factors that are perceived to influence their interprofessional and interorganizational relationships. Midwifery is a new profession in the Quebec healthcare system, nevertheless, women’s tendency to choose midwifery has increased in Quebec [22]. Therefore, to meet women’s preferences and their choice of midwifery care, and to serve the needs of women and their families, it is required that the maternity care providers work at the highest level of collaboration. The findings of our study show that maternity care professionals acknowledged that collaboration is an important aspect of care delivery. Most of our participants concurred on the meaning of collaboration as: *“working together for the same goal.”* Similar to our study, in Reiger’s study (2009), midwives perceived collaboration as a very large concept, which indicates a process of working together toward shared goals [23].

Our study demonstrated that the lack of sufficient time to collaborate and the work overload at the hospital were obstacles to interprofessional teamwork. Most of the professionals met only if they had to transfer a client or an issue for consultation that needed to be discussed. It is obvious that in such conditions, most of the time the conversations and interactions were more restricted and limited to problem solving. Previous studies showed similar results as our study [24, 25].

Our findings showed that in spite of obstetricians’ interest in integrating midwifery professionals into the hospital, midwives were skeptical and reluctant to follow this idea. Given the fact that obstetricians may create hierarchical relationships with midwives, it may place the midwives in a subordinate position. Evidently, such role changes might create a source of conflict and be a barrier to collaboration. On the other hand, our results showed some degree of conflict between midwives and obstetricians over professional philosophy, autonomy, professional territory and work style. Our nurse and family physician participants revealed the fact that the obstetricians exert power on the obstetric unit and control over almost everything, even the number of births that should take place by family physicians. Similarly, in Peterson’s study (2007) midwives described their experiences of power imbalance as “ultimate decisions are made by the obstetrician” [1].

A pilot project designed to evaluate the practice of midwifery in Quebec showed four main reasons explaining the limited integration of midwives into the maternity care system. These reasons were: the lack of knowledge about the practice of midwifery, deficiencies in the legal and organizational structure of the pilot projects where experimentation took place, competition over professional “territory”, and interpersonal gaps between midwives and other healthcare providers [26].

In our study, the nurse participants had collaboration with midwives when transferring a patient to the hospital; however, they manifested their reluctance to work collegially with midwives. The nurse participants felt that midwives might replace the nurse within maternity care and undertake many of their tasks. Similar to our study, Kornelsen’s study (2003) on the interprofessional relationships between registered midwives and perinatal nurses in British Columbia showed that even if the nurses and midwives began working together, the nurses had a negative view of midwifery practice and experienced insecurity as a result of the introduction of new midwifery professionals [27]. In New Zealand, midwifery is a deep-rooted profession and 75% of women choose a midwife as their care provider. Nevertheless, there is still little information about the quality of transfer and handover responsibility care from midwifery to obstetrics or the quality of the collaboration between midwives and obstetricians. Skinner et al. study (2010) in New Zealand showed that most of midwives (72%) felt that they had successful collaborative relationships with obstetricians, however, they felt there was still room for improvement. The consultation rate with obstetricians was 35 and 43% of women had their care transferred to an obstetrician. Midwives well supported by the obstetricians to continue care for 74% of transferred women, but a quarter of the midwives considered the collaboration was not excellent, and 14% did not feel supported by obstetricians [28]. Further work is needed to describe what successful collaboration is and how it might be implemented.

Kornelsen revealed a conflict between physicians and midwives (62%), and between nurses and midwives (60%) [27]. Mckendry revealed the struggle between professionals in Alberta, where midwives and nurses had conflict over birth attendance [29]. Kennedy and Lyndon’s study (2008) exposed the nature of nursing-midwifery relationships as both tension and teamwork. Conflicts over philosophy, respect, communication and pain management were shown to be significant between the midwives and the nurses [30]. Balis’s study (1994) on relationships between midwives and obstetrical nurses in Quebec showed that 44% of hospital nurses and 12% of community nurses believed that the arriva1 of midwives would represent a threat to the role of the nurse. Moreover, all nurses considered midwives as more of a threat to the physicians than to themselves [15]. In contrast, in developing countries such as Haiti, task shifting that recommended by the World Health Organization showed an increased access to care and solved the shortage of maternity care providers. The purpose of shifting tasks is to share the care from one level of caregiver to another to increase access to care. By training nurses and auxiliary nurse-midwives and their collaboration successfully, skilled care is delivered to women and infants in low-resource areas in Haiti [31]. Previous study on obstetrical nurses’ intentions toward collaborating with midwives in Ontario, Canada, showed that the nurses had positive intentions to collaborate with midwives, however, behaving collaboratively needed to be supported by indicating positive outcomes of collaboration and increasing inter-professional interaction [32].

Our midwife participants expressed their concern about protecting the autonomous practice in the “maison de naissances” because of maximum protection of a physiological birth and respect for women who choose to give birth out of hospital. In Peterson study (2007) the territory or “turf protection” was the term commonly used by professionals to describe the characteristic of maternity care in Canada and its interdisciplinary competitiveness. Territory protection was expressed mostly because of fears of loss of autonomous practice, loss of income, and lack of knowledge about scopes of practice in other disciplines [1]. Similar to Peterson’s study [1] the midwives in our research stated fears of losing their autonomy and their territory if they accept integration into hospitals as employees. Newnham et al. study showed although some midwives seemed reasonably comfortable working surrounded by the hospital culture, others felt in pressure as hospital culture interfered with their own philosophy [33].

Our study has some limitations. We chose a single case study as we considered the difficulty in ensuring access to maternity care professionals and administrators across different organizations. On the other hand, the time and budget available to investigate this research was limited to three years. Considering these limitations, we did not chose a multiple case study design that might have highlighted other factors that influence the collaboration between maternity care professionals in Quebec. Still, we find this research has enough strength and validity, as we deliberately chose our sample to ensure its comprehensiveness. Additionally, we applied different methods for collecting data and performed triangulation to avoid bias in data interpretation and to increase the probability of credible findings.

### Implications of the study

With this newfound information, the managers and decision-makers involved in the integration of health policies will be able to point out the factors that need to be discussed further to achieve a better maternity care service for women. The power structures and the need for midwives to be involved in policy work as well as differences in birth philosophy were important issues addressed in our study and could be instructive, influencing maternity sector change at various levels. This study results would be useful to clinicians, obstetricians and gynaecologists, midwives, nurses and all health providers in general. They will be better informed about personal and organizational obstacles and facilitating factors towards establishing more solid collaboration work. The results will assist health care providers to promote and organize mutual participation between them and the women, from the beginning of a pregnancy until its end. Considering that all organizations are faced with the challenges of growth, development and effectiveness, this contextual model, can help managers and leaders to develop proposals, to make their organisation more effective. The results also will be interesting for the Ministry of Health of Quebec, for stakeholders and decision makers to recommend how to reorganize prenatal and labour care programs at hospitals or birth centers for better quality of care. By ensuring better interprofessional and interorganizational collaboration between midwives and other health care professionals at hospitals, women would receive maternity care respecting the level of risk of their pregnancy and delivery as well as their expectations. The proposed conceptual framework could be used as a theoretical basis for future studies and could be examined in other hospitals and affiliated birth centers.

## Conclusion

Maternity care professionals are required to adapt a collaborative approach and to redraw the boundaries of responsibility. Interprofessional collaborative work between midwives and other maternity care professionals is crucial to meet of women’s needs and respect their choices. Although having collaborative and multidisciplinary teamwork is a primary goal of maternity care systems, it is difficult to achieve. The professional rivalries and philosophical differences over childbirth practice generate significant tensions in the clinical setting. A culture of interprofessional collaboration and co-operation between midwives and other maternity care professionals is beneficial to the healthcare of mothers and children, minimizing duplication of tasks and increasing job satisfaction.

## Additional file

Additional file 1: Question sheet Semi-Structured Interview Guideline. (DOCX 26 kb)

## Abbreviations

CLSC: Local community services centre
CSSS: Centre de Santé et de Services Sociaux
