# Supplementary material for: Understanding factors affecting collaboration between midwives and other health care professionals in a birth center and its affiliated Quebec hospital: a case study
Source: BMC Pregnancy Childbirth. 2017 Jun 26;17:200. doi: 10.1186/s12884-017-1381-x (PMC5485546; doi:10.1186/s12884-017-1381-x)
Supplement: Additional file 1: — Question sheet Semi-Structured Interview Guideline. (DOCX 26 kb) [file 12884_2017_1381_MOESM1_ESM.docx]

**Semi-Structured Interview Guideline**

**Participant’s number …**

**Date and hour…**

**Duration of interview...**

- Could you please describe your work experience since you have started your career as (….) and specifically since you have started working in this hospital (or birthing home), “who is doing what, with what means, toward what ends?”
- Do you consider your interaction with other maternity care professionals in the birthing center and/or hospital as a collaborative relationship?
- In your opinion, when are interactions between midwives and other maternity care professionals collaborative?
- What do you think is important for this collaboration?
- What do you experience as unpleasant regarding this collaboration?
- What are the factors that facilitate or impede the way towards a better inter-professionals and inter-organizational collaboration?
- Which action should be taken by managers, decision-makers and professionals of hospitals and birthing canter, in order to support an effective collaboration and practice of midwifery professionals in Quebec?
- If a conflict situation arises with someone you have to collaborate with, how do you deal with the situation?
- What do you think about power, power differences, power imbalances or equality between professionals, in your work place and their effects and consequences on the collaboration with other professionals?
- Are there any specific health professionals’ philosophies, values and basic theoretical perspectives inherent to your professions, or any tendency to maintain professional territories that might have an impact on the development of collaboration?
- Are there appropriate coordination and communication mechanisms in your workplace in order to develop collaborative practices among maternity care professionals?
